# Combining isotope ratios for provenancing Viking Age iron artefacts in the British Isles: a pilot study

**DOI:** 10.1039/d3ra06367d

**Published:** 2023-10-27

**Authors:** Stephen E. Harding, Chas Jones, Jane Evans, Jean Milot, Michelle Cutajar, Elizabeth Bailey, Vanessa Pashley, Doris Wagner, Peter Halkon, Mark Pearce

**Affiliations:** a National Centre for Macromolecular Hydrodynamics, School of Biosciences, University of Nottingham Sutton Bonington LE12 5RD UK steve.harding@nottingham.ac.uk; b Wirral Archaeology CIC Old Shippon, Poulton Hall, Wirral CH63 9LN UK; c Fulford Battlefield Society Parish Hall, School Lane York YO10 4LR UK chasjones1066@gmail.com; d Centre for Environmental Geochemistry, British Geological Survey Keyworth Nottingham NG12 5GG UK; e Field Museum of Natural History 1400 S Lake Shore Dr Chicago IL 60605 USA; f School of Biosciences, University of Nottingham LE12 5RD UK; g Dept. of Classics and Archaeology, University of Nottingham University Park Nottingham NG7 2RD UK mark.pearce@nottingham.ac.uk; h Emeritus Fellow University of Hull Hull HU6 7RX UK; i Honorary Research Associate, Department of Archaeology, University of York King's Manor York YO1 7EP UK

## Abstract

Stable and radiogenic isotope analysis – particularly using lead isotope analysis (LIA) – has previously been shown to be a useful tool for the provenancing of ancient metal artefacts of silver and copper and its alloys, but less progress has been made in the provenancing of iron artefacts, despite their importance and frequency in the archaeological record. In this pilot study we investigate for the first time the possibilities of iron isotope analysis in combination with trace strontium isotope analysis and LIA for the provenancing of iron objects believed to be from the Viking Age in the British Isles. Previous studies have shown that analysis of each of these isotopes can contribute to provenancing iron artefacts, but they are not individually resolutory. In this proof-of-concept study, we examine the Fe, Sr and Pb isotopes of 7 artefacts believed to derive from the Viking Age: 3 from Meols – a former Viking seaport on Wirral and 4 samples from the probable location of the AD 1066 Battle of Fulford in North Yorkshire. We also examine an additional artefact of unknown antiquity from Bebington Heath – a possible location of the AD 937 Battle of Brunanburh. Although the pilot data set is too small to make definitive conclusions, it has paved the way for a fuller study involving 100 samples (including 30 from the former Viking camp of Torksey, Lincolnshire) funded by the NEIF fund of the UK National Environmental Research Council. The high range of ^87^Sr/^86^Sr values in the present data set of 8 is beyond what would be expected for bog iron (with a cut-off ∼ 0.709) and suggests that mined ore was being used, a preliminary conclusion supported by the narrow range of Fe isotope data.

## Abstract 2

In this pilot study we investigate for the first time the possibilities of iron isotope analysis in combination with trace strontium and lead isotope analysis for provenancing iron objects believed to be from the Viking Age in the British Isles.

## Graphical abstract

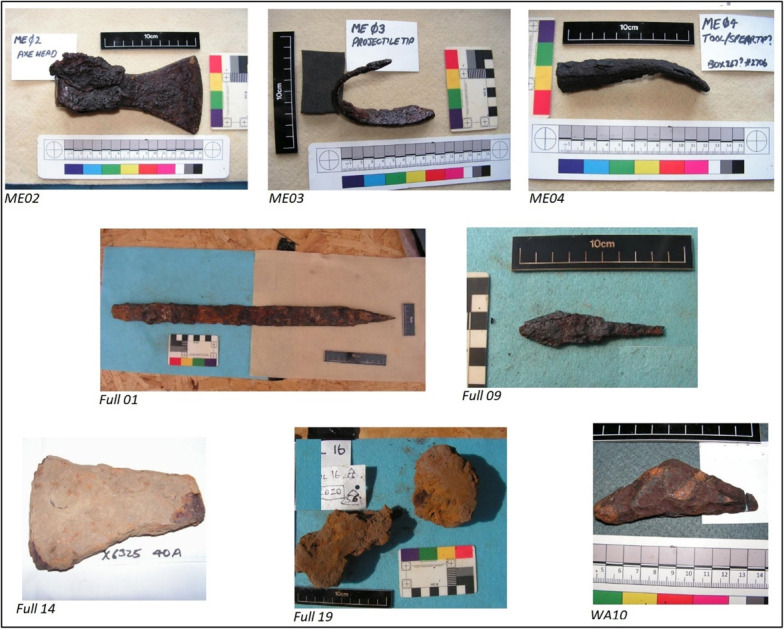

## 1. Introduction

This pilot study explores the potential of a combination of stable and “radiogenic” isotopes – the stable end products of radioactive decay series – of iron (Fe), lead (Pb) and strontium (Sr), present in iron artefacts, to provenance iron objects from the Viking Age.

Isotope analysis for provenancing is based on the principle of the measurable variation of isotope ratios of elements which are transmitted from the source to the metal. The four stable isotopes of iron are ^54^Fe, ^56^Fe, ^57^Fe and ^58^Fe with respective natural abundances of 5.8%, 91.7%, 2.2% and 0.3%. The variation of ^56^Fe/^54^Fe and ^57^Fe/^54^Fe ratios relative to an international standard may be used to fingerprint different iron sources for comparison with ancient objects. The four naturally occurring isotopes of strontium are non-radiogenic ^84^Sr, ^86^Sr and ^88^Sr, and radiogenic ^87^Sr derived from the radioactive decay of ^87^Rb. The ratio ^87^Sr/^86^Sr can be used to determine the source of various archaeological artefacts.^1^ Lead has four isotopes: stable ^204^Pb and radiogenic ^206^Pb, ^207^Pb and ^208^Pb produced by the radioactive decay of ^238^U, ^235^U and ^232^Th, respectively, with different rates of radioactive ingrowth. These characteristics make Pb a powerful tracer for archaeological artefacts.^2^

The provenancing of archaeological iron artefacts is not only useful for understanding the source of the raw materials, but also, by extension, for understanding trade routes or the migration of the people who carried them. Until recently, however, the focus has tended to have been on copper, copper alloy and silver artefacts^2^ despite the large numbers of iron artefacts found on many sites of archaeological importance. This is because lead isotope analysis (LIA) has shown itself to be a very effective tool for the provenancing of ancient metal artefacts of copper and its alloys, silver and lead^2^ but its usefulness for the provenancing of iron artefacts has not been fully established.^3,4^

Recent research has proposed the use of LIA together with trace element patterns of slag inclusions,^3^ LIA in combination with strontium (Sr) isotopes,^4^ or an alternative combination of osmium (Os) and Sr isotopes.^5^ Moreover, it has recently been proposed that Fe isotopes – based on the natural variability of iron ores – previously considered for geological or biomedical applications,^6–9^ may be useful for provenancing iron artefacts,^10–12^ particularly when used in conjunction with trace element analysis.^12^ The methodology has been successfully applied to elucidating the possible origins of 12 Roman iron bars – dating from between 100 BC to AD 100 – discovered in a shipwreck off the coast near Les Saintes-Maries-de-la-Mer (Bouches-du-Rhône), in south-eastern France.^11,12^

The main limitation for all isotopic and elemental tracers is the potential overlaps of composition between distinct sources. Combining several tracers whose variations are not correlated, as we do here, can provide complementary information (geological origin, nature of the source), and is therefore the most promising approach. One of the great advantages of isotopic analysis when dealing with archaeological materials is that it causes very little damage to ancient objects, compared to the conventional approach of trace element analysis of slag inclusions.

The approach taken by previous iron-provenancing studies has been to concentrate on analysing material from a single archaeological site and the ores which are thought likely to be the source of the iron used to make them^3–5^ rather than comparing artefacts from a selection of archaeological sites. Conversely, our pilot study takes the approach of examining material from three different archaeological sites. Iron ores are extremely widespread throughout the British Isles^13^ and consequently in many cases it is difficult to identify the likely sources of the iron ore used at specific archaeological sites, especially in later periods when iron circulated more widely.

In the British Isles, after the collapse of Roman Britain in the 4^th^ century AD, it is thought that iron was initially obtained from bog iron – which consists primarily of iron oxyhydroxides, commonly goethite (FeO(OH)) – but that it was increasingly replaced by mined iron ore in the later part of the Anglo-Saxon period and into the Viking Age.^14^ Bog iron is a form of impure iron deposit that precipitates in bogs or swamps as a result of the chemical or biochemical oxidation of iron carried in solution.

A further driver of this pilot study has been the desire to answer important questions concerning the origins of iron artefacts found in close proximity to each other at the former Viking Age seaport of Meols on Wirral, north-west England and the precise location of two Viking Age battles (Fulford AD 1066 and Brunanburh AD 937).

### 1.1. Meols, Wirral

In AD 902, Irish Chronicles known as the three fragments describe a settlement of “mass migration proportions” in Wirral, a small peninsula between Wales and Liverpool in north-west England, of Norse Vikings expelled from their former base of Dublin in Ireland.^15,16^ This was a peaceful settlement and was the result of an agreement between the Norse leader Ingimund, and Aethelflaed, Queen of the Mercian English. The peninsula is full of Norse names such as Thingwall (Old Norse *þing völlr* – “Assembly Field”) in the centre and its seaport at Meols (Old Norse *melr* – “sandbank”). In the 19^th^ century during very low tides, a large number of Viking Age metal artefacts were found and have subsequently been catalogued^17^ – our hypothesis is that isotope analysis can help provenance the iron used to make these objects, including objects from what appears to be a burial (shield boss, bent spear/projectile heads and an axe-head).^15–19^

### 1.2. Brunanburh, AD 937

The location of Brunanburh has been debated for centuries:^20^ it was the site of a key battle between a combined army of the Anglo-Saxon kingdoms of Wessex and Mercia, and a northern alliance of Scots and new wave of Norse Vikings coming from Ireland, with later reports of Icelandic Vikings fighting on the side of the Anglo-Saxon forces. It was a battle for the domination of Britain, to decide whether Britain was to be united under a single power – under the Saxon leader Aethelstan (nephew of Aethelflaed) – or would remain as discrete entities (a question which remains with us today).

The primary evidence for its location is based on place-names: Brunanburh (Brunburgh) is the old name (until the 18^th^ century) for Bromborough on the Wirral peninsula^15,20^ and in 2004 another crucial piece of evidence was elucidated: the Dingesmere mentioned in the Anglo-Saxon poem about the battle was identified as Thingsmere – *þingsmere* – the “mere” or wetland/waterway overlooked or controlled by the Viking Assembly or “Thing” (Old Norse *þing*).^15,20–22^ As a consequence, many scholars believe that the battle of Brunanburh was fought on the Wirral peninsula: a site for the battle (Bebington Heath) and Dingesmere (the River Dee coastline around Meols) has also been suggested. Increasing numbers of Viking Age artefacts are being found at Bebington and if isotope analysis can confirm that a significant number originated from Scotland, this would identify Brunanburh with respect to the other conflicts between Vikings and Anglo-Saxons that took place in the area.

### 1.3. Fulford, AD 1066

Fulford (North Yorkshire) was the location of a battle in AD 1066 between Norse invaders and Anglo-Saxons, immediately before the better known battle of Stamford Bridge. The archaeological material consists of iron objects found by excavation of a number of short-lived iron-recycling sites that were abandoned by the Norse victors at Fulford when they were defeated at Stamford Bridge five days later.^23,24^ The Fulford evidence suggests that serviceable weapons were gathered and removed while damaged metal artefacts were processed, leading to the hypothesis that the finds from the site attest to post-battle metal recycling. However, the interpretive value of scattered battlefield finds is currently limited by the available science. If it was possible to provenance or simply profile finds this might provide a link to the combatant's source of weaponry, and possibly associate the found battle fragments to help confirm battle descriptions.

### 1.4. The pilot project

**Fig. 1:**
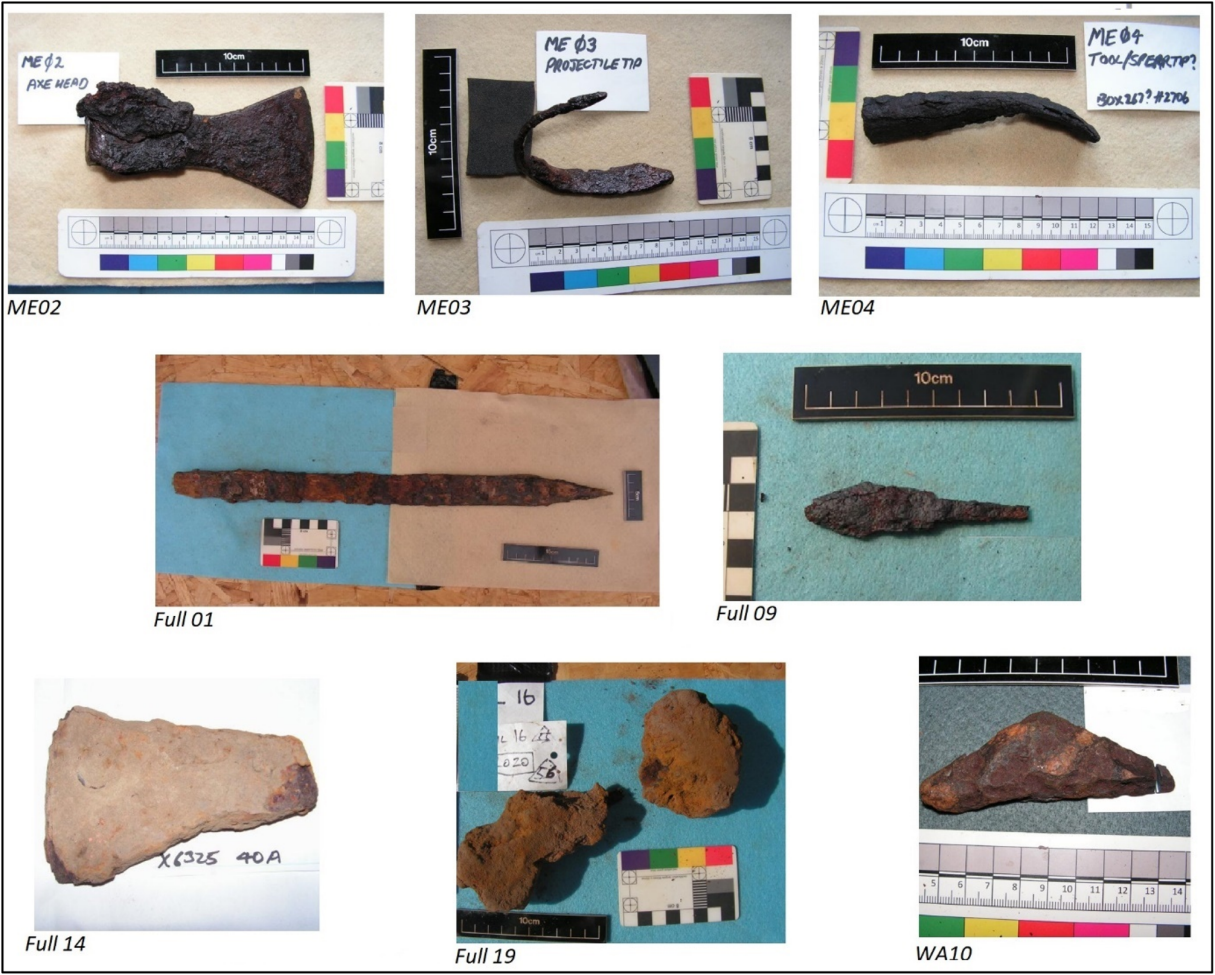
Iron objects analysed: Viking Age objects from Meols on Wirral, courtesy of the Grosvenor Museum, Chester (an axe-head ME02, a bent spearhead ME03 and a tool or spearhead ME04); Viking Age objects from Fulford, York, courtesy of the Fulford Battlefield Society (a repaired sword Full 01, a tanged arrowhead Full09, an axe-head Full14 and a planishing anvil Full19); artefact from Bebington on Wirral, courtesy of Wirral Archaeology CIC (WA10).

The considerable advances in instrumentation for analysis of isotope ratios, together with the development and population of databases,^25^ fuelled by a recent joint meeting of the Royal Society of Chemistry with the Society of Antiquaries,^25^ now makes resolution of these and other related issues a realistic possibility. For this pilot study we examined seven artefacts believed to be from the Viking Age (Fig. 1, Table 1): three from Meols on Wirral, courtesy of the Grosvenor Museum, Chester, and four from Fulford, near York, courtesy of the Fulford Battlefield Society. As a working hypothesis, it is necessary to assume that weapons/objects were made from ‘first-generation’ iron, forged from a single furnace-bloom. Conversely, if metal from different places was melted and mixed together, then provenancing becomes more difficult as the isotope signatures of the artefacts will not correspond to those of the individual ore sources. We also examined an additional artefact of unknown antiquity from Bebington Heath (also Wirral) – a possible location of the AD 937 Battle of Brunanburh – courtesy of Wirral Archaeology CIC (Community Interest Company).

**Table 1:** Sample sites for the objects

Ref.	Item description	Sampling site
ME02	Axe head	Interior of axe-shaft hole, a suitable uncontaminated site
ME03	Bent spear/projectile end	Midway surface had lifted – contained some rust and possible conservation material but solid ferrous confirmed with magnet
ME04	Spearhead shape	Section of head lifted – contained possible conservation material but solid ferrous confirmed with magnet
Ful 01	Repaired sword examined with CT scan and X-ray	Metal is visible in many places: it can be identified by its fibrous structure. Parts of surface thought to be mineralised leather (*i.e.* not metal) avoided
Ful 09	Complete conserved tanged arrow	One side of conserved surface was prised away to reveal arrow metal base below for sampling
Ful 19	Planishing anvil	Where shaft enters boss as the whole surrounded by concretion
Ful 14	Axe billet	Outer layer of metal below rust crust
WA10	Bebington Heath artefact	Non specific

## 2. Experimental section

### 2.1. Materials

The three objects from Meols, Wirral (Fig. 1) were found in close proximity to each other at low tide in sand. The four from Fulford were found by excavation. The additional wrought iron object of unknown antiquity found at Bebington Heath, also on Wirral, was also found by metal detectorists. It was originally thought to have been a pommel from a sword but subsequent X-ray analysis^26^ has shown this not to be the case. We refer to it simply as a Bebington Heath “artefact”.

### 2.2. Drilling and dissolution

A small area of each of the artefacts was abraded clear of weathering/corrosion using a diamond burr and then each sample was drilled, in order to obtain a sample of clean iron (30–50 mg for each object). To minimize the risk of damage to the objects, samples were held against a resilient pad and a handheld drill was used. Each extracted sample was split in half; one aliquoted for Fe isotope analysis at the GET laboratory (Toulouse, France) and the remainder for Sr and Pb isotope analysis at the British Geological Survey.

Aliquots dedicated to Fe isotopic analyses were weighed in clean Teflon beakers and digested using a mixture of bi-distilled 6 M HCl and 15 M HNO_3_, together with Merck supra-pure HF acid on a hot plate at 120 °C. Samples were then taken to dryness and re-digested in distilled 6 M HCl at 120 °C until no solid particles remained in the solution. Once totally dissolved, the Fe content of the samples was purified in a single step chromatography on an anion exchange Biorad© AG1-X4 resin in HCl medium.^27^

The samples for Sr and Pb analysis were transferred to a clean laboratory (class 100, laminar flow). 30–100 mg of ^84^Sr tracer solution was added depending on sample size, dissolved in Teflon distilled 8 M HNO_3_. After evaporation to dryness, the samples were converted to bromide form by addition of 0.5 M Ultrapur© HBr. Lead was collected using Eichrom© AG1X8 anion resin. The residue from this separation was evaporated to dryness and converted to chloride form by addition of Teflon© distilled 6 M HCl. The strontium was collected from this fraction using Eichrom© AG50 X8 resin.

### 2.3. Analytical methods

#### 2.3.1. Fe isotopes

After purification, the Fe isotopic composition of the samples was determined by high resolution multicollector inductively coupled plasma mass spectrometry following the procedure described by Poitrasson^28^ and Milot *et al.*^10^ The Faraday cup configuration is given in Table 2(a). In addition to ^54^Fe, ^56^Fe and ^57^Fe, the isotopes ^53^Cr, ^60^Ni and ^61^Ni were measured to correct the Cr isobaric interference on mass 54, and for mass bias correction with Ni isotopes. Each sample was bracketed by analysing IRMM-14 reference material in the analytical sequence. The mass bias was corrected by combining standard-sample bracketing and by a daily regression method using the Ni added in every sample and standard solutions. In addition, we measured the composition of an in-house haematite standard (ETH haematite from Milhas, Pyrenees Mountains, France) every six samples for quality control. The Fe isotopes composition of the samples and standard are expressed in delta notation,^10^ in per-mil (‰), relative to IRMM-14 (for example for ^57^Fe/^54^Fe ratio: δ^57^Fe = {(^57^Fe/^54^Fe)_Sample_/(^57^Fe/^54^Fe)_IRMM-14_ −1} × 1000). Each sample was analysed at least three times (*n* = 3 for ME04, WA10, FUL01, FUL09, FUL19, and *n* = 6 for ME02, ME03 and FUL14) and the analytical uncertainties in Table 3 are reported as 2SE (standard error).

**Table 2:** Faraday cup configuration for (a) Fe isotope measurement (b) Sr isotope measurement and (c) Pb isotope measurement

Faraday cup	L4	L3	L2	L1	Ax	H1	H2	H3	H4
**(a) Fe isotope analyses**									
Measured element			^54^Fe	^56^Fe		^57^Fe			
Mass bias correcting element							^60^Ni		^61^Ni
Isobaric interference	^53^Cr^a^		^54^Cr						

**(b) Sr isotope analyses**									
Measured element (multidynamic measurement on Triton thermal ionisation mass spectrometer)		^84^Sr		^86^Sr	^87^Sr	^88^Sr			
			^86^Sr	^87^Sr	^88^Sr				
		^86^Sr	^87^Sr	^88^Sr					
Mass bias correcting element	Corrected using multidynamic algorithm with ^86^Sr/^88^Sr = 0.1194								
Isobaric interference			^85^Rb		^87^Rb				

**(c) Pb isotope analyses**									
Measured element				^204^Pb		^206^Pb	^207^Pb	^208^Pb	
Mass bias correcting element			^203^Tl		^205^Tl				
Isobaric interference		^202^Hg^a^		^204^Hg					

a ^53^Cr and ^202^Hg were measured for the correction of the isobaric interference of ^54^Cr on ^54^Fe, and ^204^Hg on ^204^Pb, respectively.

Because of the mass dependent fractionation of iron isotopes in nature (δ^57^Fe = δ^56^Fe × 1.5), the use of δ^57^Fe or δ^56^Fe makes no difference for discussing the results. However, we preferentially report δ^57^Fe in the discussion part of this paper as it yields greater variation than δ^56^Fe because of the 3 atomic mass units difference between ^57^Fe and ^54^Fe. The 21 measurements of the ETH haematite displayed δ^57^Fe of (0.761 ± 0.082)‰ and δ^56^Fe of (0.517 ± 0.060)‰ (2SD). This is consistent with previous measurement of the same ETH standard reported by Sossi *et al.*^29^ who found δ^57^Fe = (0.753 ± 0.094)‰ and δ^56^Fe = (0.514 ± 0.049)‰ and Ratié *et al.*^30^ who found δ^57^Fe = (0.762 ± 0.083)‰. This indicates the good quality of our results.

#### 2.3.2. Sr isotopes

Strontium was loaded onto a single Re filament following the method of Birck^31^ and both the isotope composition and strontium concentrations were determined by Thermal Ionisation Mass Spectroscopy (TIMS) using a Thermo Triton (Thermo Scientific, Bremen, Germany) multi-collector mass spectrometer, with Faraday cup configuration given in Table 2(b). The international standard for ^87^Sr/^86^Sr, NBS987, gave a value of 0.710 259 ± 0.000020 (2SD, *n* = 8) during the analysis of these samples and the data are normalised to the accepted value of 0.710 250. The international rock standard (Columbia River Basalt BCR-2) gives the following reproducibility through sample dissolution, column separation and mass spectrometry analysis: ^87^Sr/^86^Sr = 0.705 016 ± 0.000026 (2SD, *n* = 26) during the analysis of the samples in this study. This compares very favourably with the accepted value of 0.705 013 ± 0.000010 (2SD, *n* = 13) – see ref. 32.

#### 2.3.3. Pb isotopes

Pb isotope analysis of the samples was conducted using a Thermo Scientific (Bremen, Germany) Neptune Plus MC-ICP-MS (multi-collector inductively coupled plasma mass spectrometer). This mass spectrometer is fitted with the Jet interface, in which enhanced sensitivity is achieved through the use of a large volume interface pump (Pfeiffer On-Tool Booster 150) in combination with the Jet sampler and X skimmer cones. Prior to analysis, each sample was appropriately diluted (using Teflon distilled 2% HNO_3_) and spiked with a solution of thallium (Tl), which is added (in a ratio of ∼1_Tl : 10_Pb: this provides an intensity that can be measured accurately while minimising the use of highly toxic thallium) to allow for the correction of instrument induced mass bias. Samples were then introduced into the instrument *via* an ESI 50 μl min^−1^ PFA micro-concentric nebuliser attached to a desolvating unit, (Cetac Aridus II). All Pb isotopes of interest were simultaneously measured using the Faraday (see, *e.g.* Evans *et al.*^33^) cup configuration detailed in Table 2(c).

The acquisition consisted of 50 ratios, collected at 8.4 second integrations, following a 60 second de-focused baseline measurement made at the beginning of each analytical session.

The precision and accuracy of the method was assessed through repeat analysis of NBS 981 Pb reference solution, (also spiked with Tl). Data are corrected (normalised) relative to the known values for this reference, taken from Thirlwall:^34^^206^Pb/^204^Pb = 16.9417, ^207^Pb/^204^Pb = 15.4996, ^208^Pb/^204^Pb = 36.724, ^207^Pb/^206^Pb = 0.91488, ^208^Pb/^206^Pb = 2.1677. The analytical errors, reported for each of the sample ratios, are propagated relative to the reproducibility of the session NBS 981, to take into account the errors associated with the normalisation process. A secondary standard, (NBS 982), with defined values of ^206^Pb/^204^Pb = 36.7432, ^207^Pb/^206^Pb = 0.467 084 and ^208^Pb/^206^Pb = 1.00016 (see ref. 35) gave ^206^Pb/^204^Pb = 36.7462, ^207^Pb/^206^Pb = 0.46715 and ^208^Pb/^206^Pb = 1.00043 after normalization to NBS981.

As with strontium isotope analysis, the international rock standard (Columbia River Basalt BCR-2) gives the following reproducibility through sample dissolution, column separation and mass spectrometer analysis for lead isotopes: ^206^Pb/^204^Pb = 18.7524 ± 0.0191, ^207^Pb/^204^Pb = 15.6272 ± 0.0050, ^208^Pb/^204^Pb = 38.7231 ± 0.0290 (2SD, *n* = 3). This compares very favourably with the published values for this standard, namely:^32^^206^Pb/^204^Pb = 18.7529 ± 0.0195, ^207^Pb/^204^Pb = 15.6249 ± 0.0040, ^208^Pb/^204^Pb = 38.7237 ± 0.04 (2SD, *n* = 11).

We plot the conventional ^207^Pb/^204^Pb *vs.*^206^Pb/^204^Pb and ^208^Pb/^204^Pb *vs.*^206^Pb/^204^Pb, ratios and we also plot the “Model Age *T* (Ma)”, where Ma means “millions of years”, *vs.* the *μ* (=^238^U/^204^Pb) parameter as described by Albarède *et al.* (2012).^36^ This latter method provides a more accessible method of providing reference fields that relate to major tectonic related mineralization events.^41^

## 3. Results and discussion

### 3.1. Fe isotope analysis

**Fig. 2:**
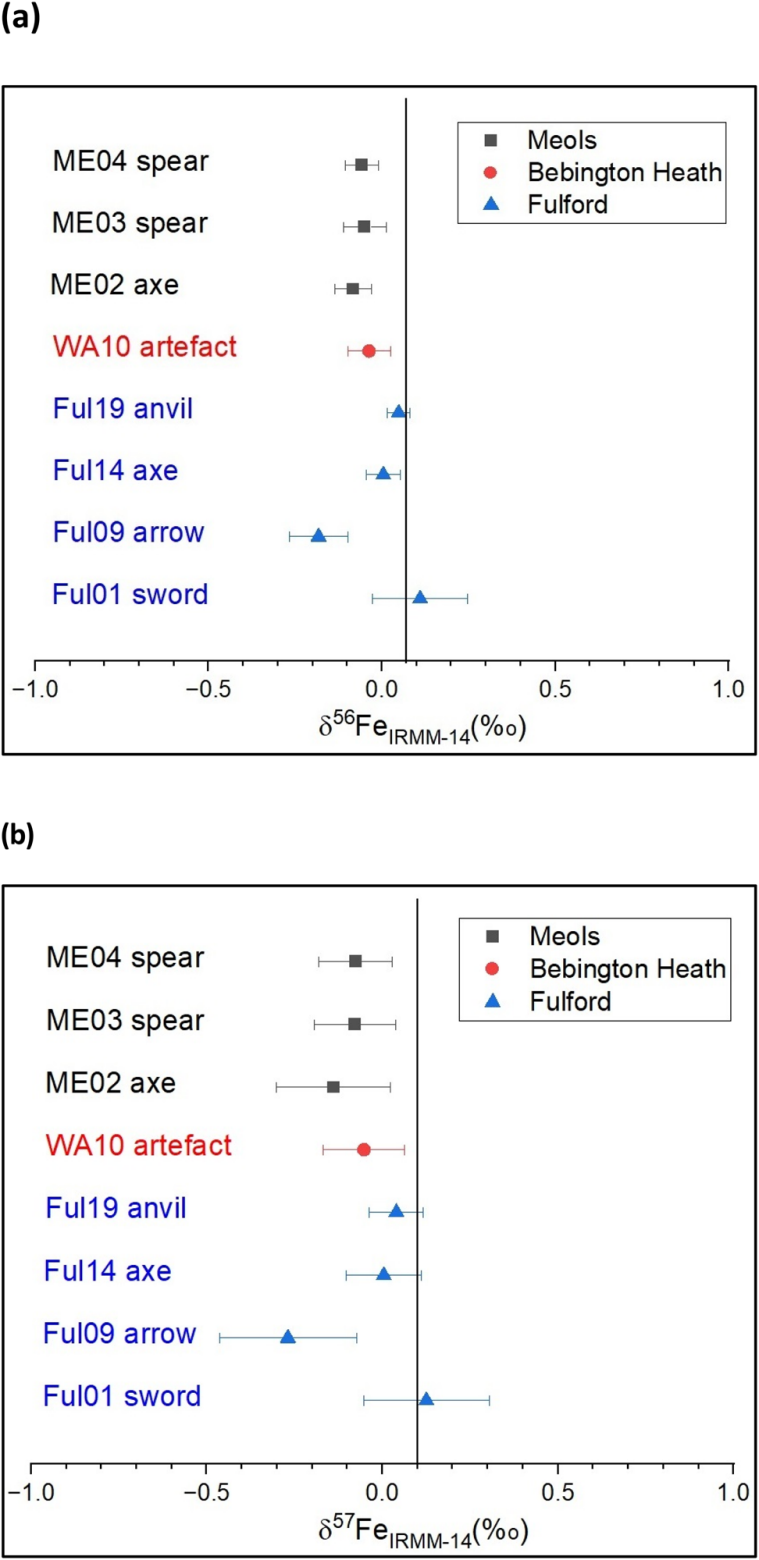
Fe isotope composition of iron objects from Meols, Bebington Heath (Wirral) and Fulford, expressed as (a) δ^56^Fe (‰) and (b) δ^57^Fe (‰) relative to IRMM-14 Fe isotopic material. The vertical lines correspond to the mean composition of Earth's crust estimated at (a) δ^56^Fe = 0.07‰ (calculated from ref. 35), and (b) δ^57^Fe = 0.1‰.^28^

Values for δ^56^Fe and δ^57^Fe are shown in Table 3, and Fig. 2. Significant variation of Fe isotopic composition appears among the 10 iron objects analysed. The samples from Meols have a very narrow composition range comprised between −0.139 and −0.076‰ for δ^57^Fe. Those from Fulford display a broader range of δ^57^Fe values, from −0.269 to 0.125‰. The Fe isotopic composition of the artefacts from Meols, which is relatively homogeneous, might be taken to indicate a single ore source, but the Sr and Pb isotope data for these objects do not show close grouping: this shows that isotope ratio data from more than one element is necessary before making robust conclusions on a common provenance of a group of objects. In contrast, the objects from Fulford display significantly distinct Fe isotopic compositions, which likely indicates that they were made of iron from a variety of ore sources.

**Table 3:** Isotope results for the pilot study^a^

	ME 02	ME 03	ME 04	Ful 01	Ful 09	Ful 14	Ful 19	WA 10
**Iron**								
δ^56^Fe (‰)	−0.083	−0.050	−0.059	0.110	−0.183	0.004	0.048	−0.037
2SE	0.052	0.062	0.048	0.137	0.085	0.049	0.033	0.062
δ^57^Fe (‰)	−0.139	−0.078	−0.076	0.125	−0.269	0.004	0.040	−0.052
2SE	0.162	0.117	0.104	0.179	0.195	0.108	0.077	0.116

**Strontium**								
Total Sr (μg g^−1^)	5.48	1.72	0.4	0.61	1.87	0.85	0.30	0.40
^87^Sr/^86^Sr	0.71005	0.71414	0.70911	0.71178	0.71534	0.71311	0.71407	0.70966
2SE	0.00002	0.00002	0.00001	0.00002	0.00003	0.00003	0.00002	0.00007

**Lead**								
^206^Pb/^204^Pb	18.1804	18.3478	18.3858	18.2732	18.4563	18.3146	18.5429	18.4030
2SD	0.0007	0.0007	0.0009	0.0016	0.0011	0.0009	0.0011	0.0009
^207^Pb/^204^Pb	15.6247	15.6400	15.6401	15.6248	15.6354	15.6250	15.6294	15.6495
2SD	0.0016	0.0008	0.0008	0.0016	0.0011	0.0009	0.0011	0.0008
^208^Pb/^204^Pb	38.1918	38.4099	38.4276	38.2565	38.4692	38.3401	38.5723	38.4616
2SD	0.0008	0.0008	0.0008	0.0008	0.0008	0.0008	0.0008	0.0008
^207^Pb/^206^Pb	0.85942	0.85241	0.85066	0.85507	0.84716	0.85314	0.84286	0.85038
2SD	0.00006	0.00006	0.00006	0.00009	0.00007	0.00006	0.00008	0.00006
^208^Pb/^206^Pb	2.10078	2.09348	2.09011	2.09365	2.08441	2.09350	2.08017	2.09001
2SD	0.00006	0.00006	0.00006	0.00008	0.00006	0.00006	0.00006	0.00006
*T* (Ma)	417	322	294	349	241	318	166	298
*μ* = ^238^U/^204^Pb	9.764	9.784	9.773	9.741	9.757	9.732	9.718	9.807

a SE: standard error; SD: standard deviation. *T* is the ‘Model Age’ parameter (in millions of years).^35^

Although important overlaps occur between the sites, a striking point is the narrow total range of isotopic variability of these 8 objects (0.421‰ for δ^57^Fe). This may indicate hydrothermal-derived ore sources for these objects, instead of sedimentary iron ores which would display more fractionated compositions.^12^ In particular, these results do not argue for a source from bog iron ores from eastern England, since the high variability previously measured in such ores (about 4‰ for δ^57^Fe measured in bog iron from Germany by Rose *et al.*^37^) would likely to have been reflected in these objects. Unfortunately, that is as much as we can say at the present time as there is a sparsity of comparative data for the British Isles and Scandinavia, although commercial analysis facilities are now available^38^.

### 3.2. Sr isotope analysis

**Fig. 3:**
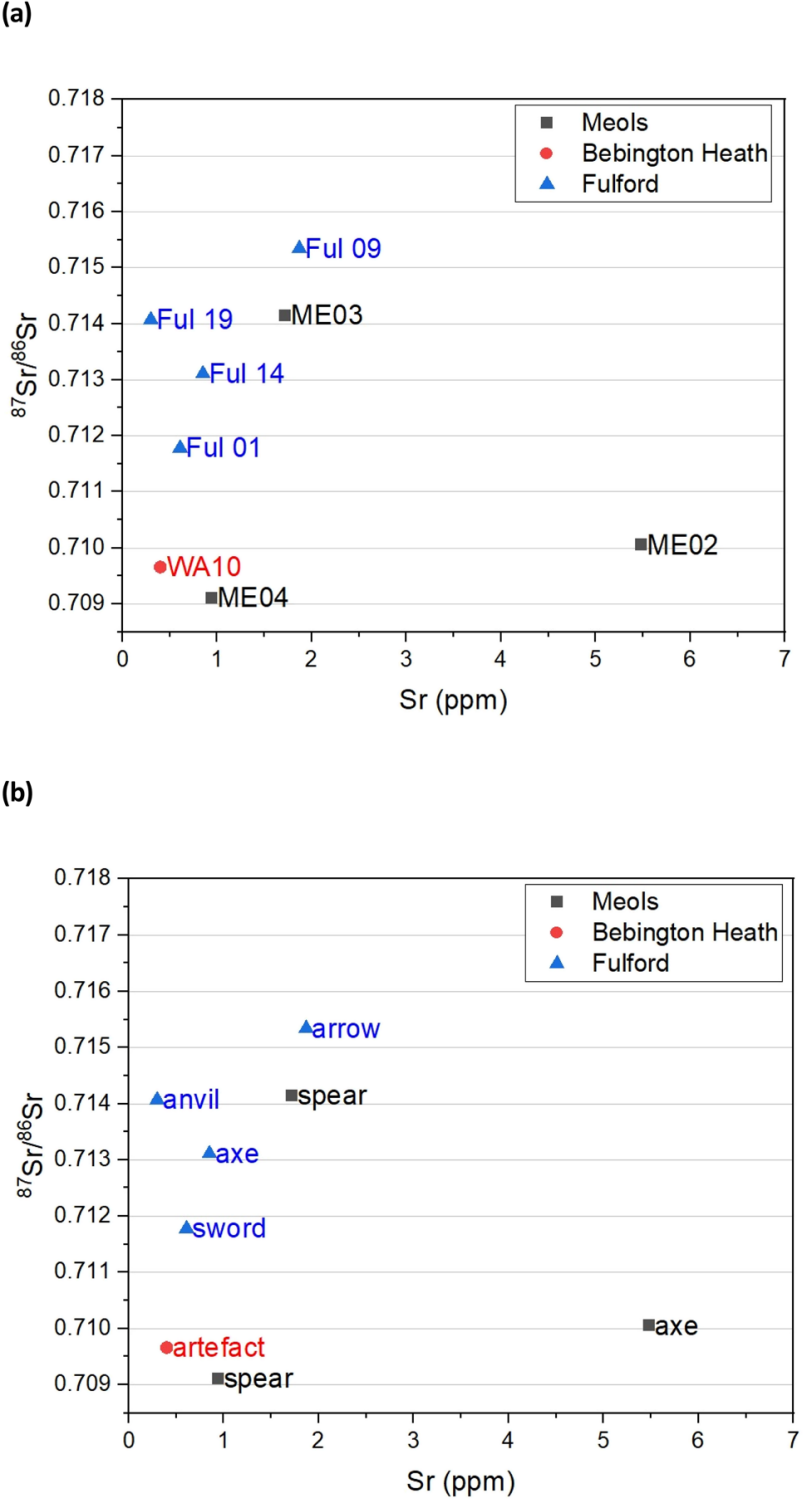
Strontium isotope analysis (a) Sr concentration plotted against ^87^Sr/^86^Sr composition for the artefacts, and (b) same plot but with the object types. The uncertainties in concentration are ±1.7% 2SD and the uncertainties in the isotope ratio are ±0.000020 2SD based on the reproducibility of NBS 987 standard. This is < the size of the symbols used.

Fig. 3 gives the distribution of the minor strontium isotope ratio ^87^Sr/^86^Sr as a function of strontium concentration for the eight iron objects. The Sr concentrations are generally low, between 0.5 and 5 ppm (μg g^−1^) similar to the range (>15 ppm) noted for iron artefacts from south-east Turkey.^39^ The Sr isotope compositions are variable and range between 0.7091 and 0.7153. There is no correlation between the Sr composition and the sites at which they were found.

There is little published data on the Sr isotope composition of iron in Northern Europe with which to compare the data from the weaponry. However, four samples have been analysed from iron production sites along the River Foulness, near Holme-on-Spalding-Moor, East Riding of Yorkshire (Table 4). All the Sr concentrations are below 0.7097. These bog iron and slag sample values are consistent with an ore formed in coastal wetland or rain supplied bogs which are dominated by marine/rainwater values close to 0.7092.^40^

**Table 4:** Strontium isotope results for bog iron/slag from River Foulness sites

Ref.	Item description	Total Sr (μg g^−1^)	^87^Sr/^86^Sr	2SE
FSS09	Slag	12.0	0.70940	0.00001
FSS10	Hearth bottom	36.3	0.70845	0.00001
FSS11	Slag & burnt stone	27.3	0.70961	0.00001
FSS12	Bog iron	51.9	0.70909	0.00001

The results from the artefacts display a far wider range of Sr values than can be accounted for from coastal wetlands and rain dominated bogs (Fig. 3b). A number of possible reasons for the data range can be posited that will be the focus of future studies beyond this pilot, namely (1) their ore comes from a geologically deposited source related to mineralization (2) that there are bogs with water sources that are not predominantly rainwater, but possible aquifer on rocks, with more radiogenic source, or (3) the process of making the weaponry involved the addition of a component with radiogenic Sr values.

### 3.3. Pb isotope analysis

**Fig. 4:**
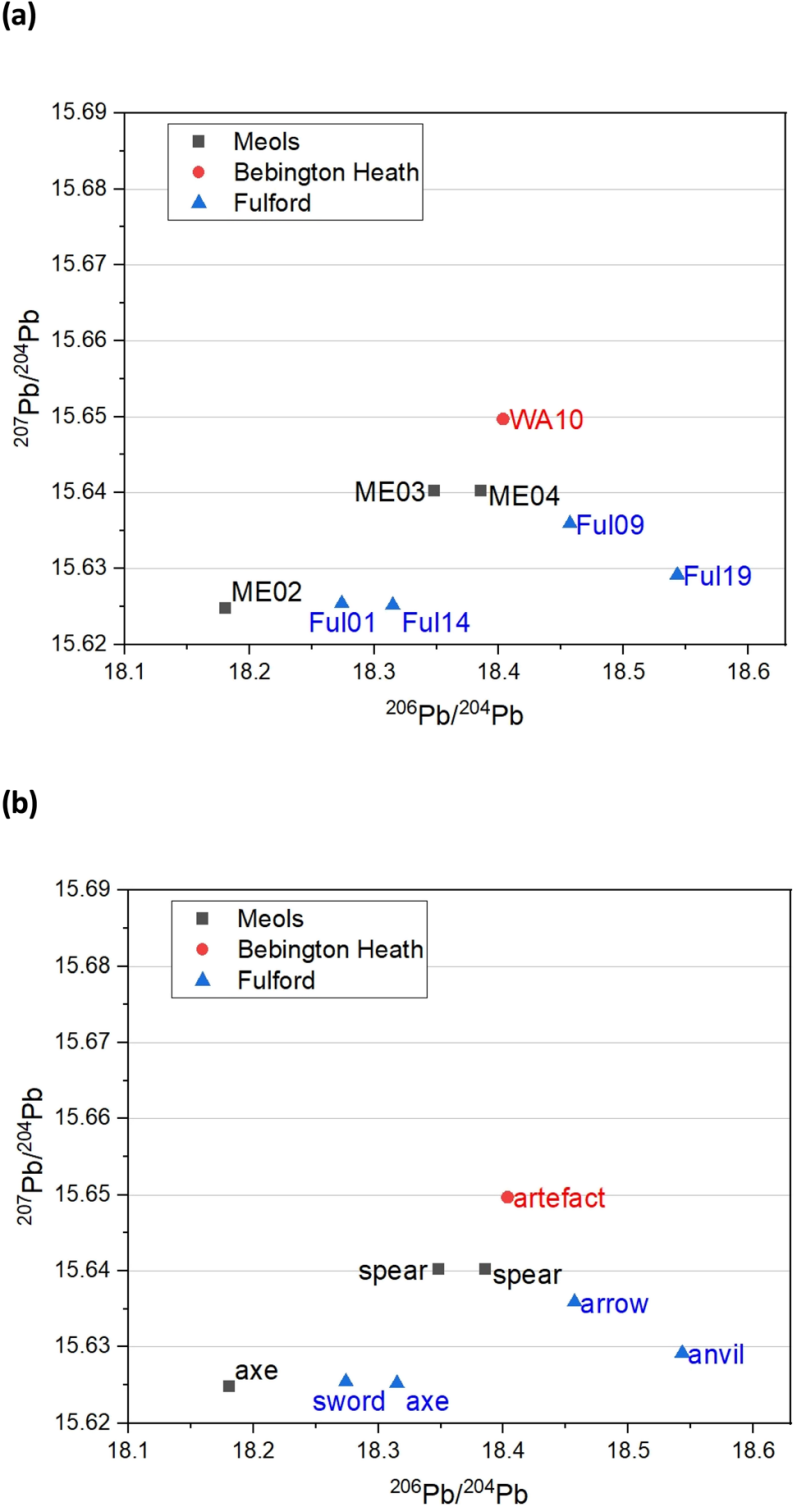
Lead isotope analysis (a) Pb isotope composition of iron objects from Meols, Bebington (Wirral) and Fulford, and (b) same plot but with the object types. Errors (±2SD): ^207^Pb/^204^Pb = ±0.0050; ^206^Pb/^204^Pb = ±0.0191.

A better picture comes from trace Pb isotope analysis of the iron objects – this is primarily because, in contrast with the Fe and Sr isotope data, the databanks of Pb isotopes for the British Isles are much better populated, based on rock, ores and faunal sources.^32,33^Fig. 4 gives bivariant diagrams of ^207^Pb/^204^Pb *versus*^206^Pb/^204^Pb minor isotope ratios for our 8 objects.

**Fig. 5:**
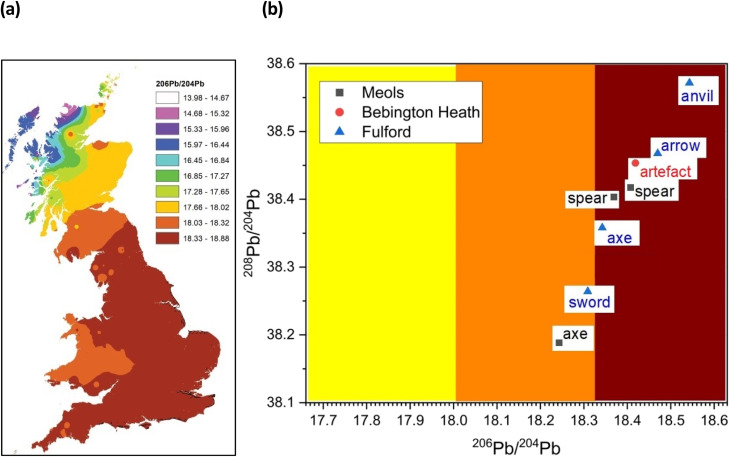
(a) Distribution of ^206^Pb/^204^Pb within Britain based on data from mineral sources. Adapted from Evans *et al.* (2022)^33^ and reference cited therein. (b) Plot of ^208^Pb/^204^Pb *vs.*^206^Pb *vs.*^204^Pb with the contour zones from (a).

**Fig. 6:**
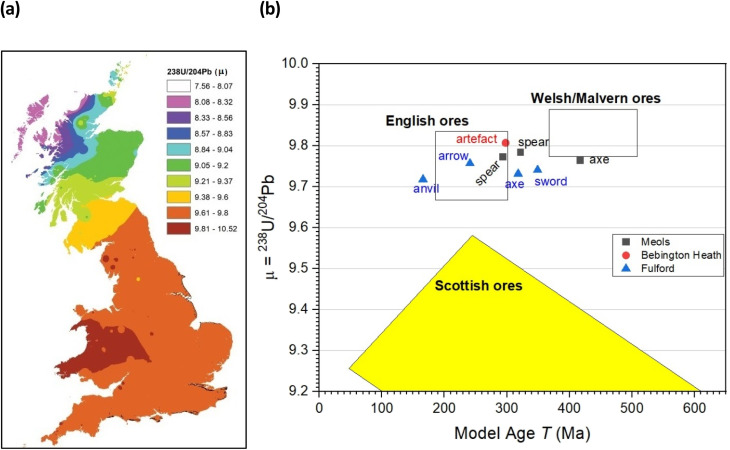
(a) Distribution of ^238^U/^204^Pb (*μ*) within Britain based on data from mineral sources. Adapted from Evans *et al.*^33^ and references cited therein (b) plot of *μ* = ^238^U/^204^Pb *versus* the Model Age parameter *T* (in millions of years).

In a recent study, Evans and colleagues^33^ explored the distribution of Pb isotopes throughout the British Isles, taking advantage of the fact that unlike for strontium isotopes whose distribution is affected by underlying rocks, for lead there is a tectonic boundary between the Solway Firth and Berwick on Tweed – the Iapetus Suture – with clear isotope signatures appearing to the north and south of the suture which closely maps the current boundary between Scotland and England. Fig. 5a shows the contoured map of ^206^Pb/^204^Pb – with distinct demarcations – and Fig. 6a shows the distribution of the related ^238^U/^204^Pb (*μ* values), which shows even greater resolving potential. Scottish Pb mineralization generally has a significantly different “older” isotope signature^39^ separated by the so-called Iapetus Suture.^41^

We can use this variation in ^206^Pb/^204^Pb or the derived parameter *μ* in one of two ways. Firstly, in terms of a bivariant plot of ^208^Pb/^204^Pb (*y*-axis) *versus*^206^Pb/^204^Pb (Fig. 5b); or equivalently following Albarède *et al.*^36^ and Evans *et al.*^33^ plotting *μ* (*y*-axis) *versus* the ‘Model Age *T*’ parameter (Fig. 6b).

## 4. Concluding remarks

The limitations of trying to make conclusions about the provenancing of 8 objects (from 3 sites in the British Isles) based on current databases are all too clear. Our pilot study shows patterns in the data, but the number of samples was much too low to understand the significance of this finding, and particularly with the iron and strontium isotope data we await the development of databases against which to compare our own samples. The high range of ^87^Sr/^86^Sr values, beyond what would be expected for bog iron (with a cut-off around 0.709), suggests that mined ore was being used, a preliminary conclusion supported by the Fe isotope data, and that the Sr in some of the samples is likely to come from sources other than bog iron. And this study cannot exclude the possibility that some of the iron objects could be derived from direct mineralization deposits rather than secondary bog precipitation: that question will not be addressed until we have more direct measurements of Sr in bog-iron samples.

The more extensive work on lead isotopes across the British Isles in particular, show clear differences in published data for Scotland due to the Iapetus Suture and the known uranium depletion of the old Laurentian basement which underlies much of Scotland.^33^ This is particularly important in trying to address the question of whether any iron objects found at Bebington on Wirral are unequivocally associated with the lost Battle of Brunanburh. The current “test” object – WA10 of unknown antiquity clearly is not, and may not be Viking Age at all. It also needs to be established what the controls from Ireland and Scandinavia are like, to enhance the diagnostic plots such as Fig. 5b and 6b, which are likely to be further refined as the databanks grow.

Nonetheless this pilot study has paved the way for a more extensive study where we continue to analyse a much larger number of objects (90) from Fulford, Bebington Heath and the former Viking army encampments of Torksey (Lincolnshire), and Aldwarke (South Yorkshire)^42,43^ together with 10 samples of bog iron and slag from the Foulness Valley (East Riding of Yorkshire). The more samples of bog iron from this latter important and well-documented^44^ source – known to have been exploited since the later first millennium BC – will be included in the extended study in order to exemplify the isotope signal of bog iron. We may then be able to start to obtain some general ideas as to the provenance of the artefacts. The work on iron, strontium and lead isotopes will also be reinforced by trace osmium isotope analysis^45^ and investigating the relative concentrations of a range of trace elements, including: phosphorus (P), manganese (Mn), barium (Ba), cobalt (Co), nickel (Ni), copper (Cu), zinc (Zn) and arsenic (As), as well as of course strontium (Sr) which has been included as part of the present pilot study. Iron, strontium, lead and their isotopes thus far seem to tantalisingly offer the best hope for separating iron provenances, but more objects need to be found and the databases need to be extended.^46^ Indeed, this work is being undertaken in parallel with growth of the appropriate databases, recently stimulated by a joint meeting of the Royal Society of Chemistry and the Society of Antiquaries (UK).^25^

## Conflicts of interest

There are no conflicts to declare.

## Supplementary Material
